# Influence of Irritable Bowel Syndrome on Stress and Depressive Symptoms in Nurses: The Korea Nurses’ Health Study

**DOI:** 10.3390/ijerph182312324

**Published:** 2021-11-24

**Authors:** Oksoo Kim, Chiyoung Cha, Hyunseon Jeong, Mijung Cho, Bohye Kim

**Affiliations:** College of Nursing, Ewha Womans University, Seoul 03760, Korea; ohong@ewha.ac.kr (O.K.); chiyoung@ewha.ac.kr (C.C.); idadoshi@hotmail.com (H.J.); amillan8@naver.com (M.C.)

**Keywords:** irritable bowel syndrome, stress, depression, female, nurses

## Abstract

Despite the high risk of irritable bowel syndrome (IBS) and stress/depressive symptoms in nurses, limited research has examined the relationship between these conditions in female nurses. The purpose of this study was to determine the prevalence of IBS and the influence of IBS on stress and depressive symptoms in female nurses. We analyzed the data from the Korea Nurses’ Health Study. Among 7667 participants from the 7th survey conducted from December 2018 to September 2019, 178 nurses were identified as having IBS based on the Rome IV criteria. Using the propensity score matching, 712 were selected as a comparison group. Multivariate ordinal logistic regression analyses were conducted to examine the influence of IBS on stress and depressive symptoms. The prevalence of IBS was 2.3% and the prevalence of depressive symptoms among nurses with IBS was 13.5%. Female nurses with IBS were 2.214 times more likely to experience increased stress levels. However, when demographics, dietary habits, sleep quality, and depressive symptoms were considered, having IBS was no longer an influential factor for increased stress levels. When all the variables were considered, female nurses with IBS were still 2.205 times more likely to experience depressive symptoms. Adequate support is needed to relieve depressive symptoms in nurses with IBS.

## 1. Introduction

Irritable bowel syndrome (IBS) is a functional gastrointestinal disorder characterized by recurrent abdominal pain associated with changes in defecation or bowel habits [1]. The Rome criteria, which were developed by the Rome committee to screen for functional gastrointestinal disorders, are used to diagnose IBS [2]. The Rome III criteria were introduced in 2006, and to accommodate new information about the epidemiology, diagnosis, and treatment of IBS, the Rome IV criteria were released in 2016 [1].

The prevalence of IBS is reported differently in each country depending on the diagnostic criteria used. In a systematic review of 83 studies from 41 countries, the prevalence of IBS varied from 1.1% in France and Iran to 35.5% in Mexico, and the mean prevalence in Asia was 9.6% [3]. In a meta-analysis of 57 studies, the global prevalence of IBS was found to be 9.2% based on the Rome III criteria and 3.8% based on the Rome IV criteria [4]. The prevalence of IBS in women is higher than in men, and by age, the prevalence is higher in young people [4,5]. The reason for the high prevalence in women is not yet clear, but it could be due to cultural factors. Also, it is known that sex hormones can affect the intestinal barrier function, immune activation of intestinal mucosa, and gut microbiota [6].

IBS is associated with psychological health problems such as low quality of life, depressive symptoms, and anxiety [7,8]. In patients with IBS, comorbidity of psychiatric disorders is high [9,10], possibly due to complex reciprocal interactions in the bidirectional connection system of the brain-gut axis [10]. It has been reported that women with IBS experience more psychological symptoms than men with IBS [6]. IBS also has a negative effect on patients’ work and social lives [7]. The economic burden of patients with IBS is high; it has been estimated that 48% of patients in the United States pay $740 per year or more for treatment [11].

Patients with IBS have higher levels of stress than healthy people [12,13]. Stress in patients with IBS was positively correlated with depressive symptoms and negatively correlated with quality of life [14]. Stress could act as a predisposing factor for IBS or appear due to IBS [15]. However, in a study of patients with IBS with diarrhea, Chan et al. [16] showed that daily life stress can reduce bowel symptoms. Therefore, studies are needed to confirm the relationship between IBS and stress.

The depressive symptoms of patients with IBS should be studied because they negatively affect psychological and social health. In a meta-analysis, Lee et al. [17] reported that the level of depressive symptoms among patients with IBS was higher than that in healthy subjects. In a systematic review, Zamani et al. [8] reported that among patients with IBS, the risk of developing depressive symptoms was three times higher than that among healthy subjects. Depressed patients with IBS had lower quality of life and higher levels of fatigue and gastrointestinal symptoms than patients with IBS without psychological distress [18]. 

Nurses are at a higher than average risk of developing IBS due to the irregular life rhythms and sleep problems associated with shift work [19]. Nurses are also one of the occupational groups with high levels of stress and depressive symptoms [20,21,22]. To safeguard the psychological health of nurses with IBS, it is necessary to examine the relationship between IBS and stress and depressive symptoms. Although the Rome IV criteria are based on new information about epidemiology, diagnosis, and treatment of IBS [1], studies using the Rome IV criteria to determine IBS prevalence are significantly fewer than those using the Rome III criteria [4]. Therefore, this study uses the Rome IV criteria to examine the prevalence of IBS among female nurses and examines the effect of IBS on stress and depressive symptoms.

## 2. Materials and Methods

### 2.1. Design

This cross-sectional study used data from an ongoing, large-scale, prospective cohort study, the Korea Nurses’ Health Study (KNHS). KNHS was launched in 2013 with the aim of identifying factors that affect the health status and diseases of women of childbearing age. KNHS uses the same protocol as the Nurses’ Health Study 3 (NHS3) in the United States (US), but the questionnaire was revised to better fit Korean situations. The study design and protocol are described in more detail elsewhere [23]. 

### 2.2. Participants

This study enrolled registered nurses who were employed at the time of data collection. The study population of the KNHS baseline survey from 2013 to 2014 comprised female nurses aged 20 to 45 years and employed at hospitals. A total of 20,613 nurses from the baseline survey were invited via text message or email to continue taking part in sequential online follow-up surveys. The number of participants in the 7th survey, from December 2018 to September 2019, was 7667. Among those 7667 participants, 178 were identified as having irritable bowel syndrome based on the Rome IV criteria. Using propensity score matching, 712 nurses from that cohort were selected as a comparison group. Therefore, the analyses here involve 890 participants.

### 2.3. Data Collection

Participants responded to the questionnaire on the KNHS website through a link in a text message, or they participated directly on the website through a personal computer. IBS, IBS-related characteristics, stress, depressive symptoms, demographic characteristics, dietary habits, and sleep quality were the variables used in this study. 

IBS was classified using the Rome IV criteria [1]: recurrent abdominal pain for, on average, at least 1 day a week for the past 3 months and associated with two or more of the following criteria: (a) related to defecation; (b) associated with a change in the frequency of stool; (c) associated with a change in stool form. The sensitivity and specificity of the Rome IV criteria used to classify IBS were 62.7%, and 97.1%, respectively [2].

Items characteristic of IBS include the bowel movement pattern and medication/health behaviors used for intestinal health. The bowel movement pattern was evaluated with the question, ‘Do you think that you have decreased bowel movements or difficulty defecating in the past 3 months?’ If participants answered yes, they were asked about their number of bowel movements.

Stress was measured using the Perceived Stress Scale-10 (PSS-10) [24]. Each item was measured on a 5-point Likert type scale, and the possible score range was 0–40 points, with higher scores indicating more severe stress levels. In this study, a score of 21 or higher (based on the 75% percentile) was defined as a high stress level. PSS-10 was confirmed to be reliable and valid by comparison with the original 14-item PSS [24]. Cronbach’s alpha was 0.76 in this study.

Depressive symptoms were measured using the Patient Health Questionnaire (PHQ-9) [25]. Each item was rated on a 4-point Likert type scale, and the possible score range was 0–27 points, with higher scores indicating more severe depressive symptoms. The level of depressive symptoms was classified as no to mild symptoms (<10) and moderate to severe symptoms (≥10) [25]. The sensitivity and specificity of the cutoff score of 10 points were both 88% [25]. In the present study, Cronbach’s alpha was 0.88.

The demographic characteristics collected were age, educational level, marital status, body mass index (BMI), alcohol consumption, and shift work. BMI was calculated based on the participants’ self-reported height in meters and weight in kilograms. BMI was categorized as underweight <18.5 kg/m^2^, normal 18.5 to <23 kg/m^2^, or overweight ≥23 kg/m^2^. Alcohol consumption was assessed by asking about drinking experience during the past 3 months with a yes or no dichotomous response. 

Items for dietary habits included the frequency of meals, amount of meal, and frequency of skipping meals. To assess meal frequency, participants were asked to give the number of meals they ate per day for the past 3 months. The amount of meal was surveyed with the question, ‘How large were your meals in the past 3 months?’ Three responses were offered: ‘small’, ‘adequate’, ‘large’. To assess the frequency of skipping meals, we asked how many meals participants had skipped per week for the past 3 months.

Sleep quality was surveyed with the question, ‘During the past month, how would you rate your sleep quality overall?’, from the Pittsburgh Sleep Quality Index [26]. Four responses were offered: ‘very good’, ‘fairly good’, ‘fairly bad’, or ‘very bad’. Because the number of ‘very good’ and ‘very bad’ responses was small, merged data were analyzed.

### 2.4. Data Analysis

Data were analyzed using SPSS version 26. Descriptive statistics were used to describe IBS-related characteristics. Differences in the demographic characteristics, dietary habits, sleep quality, stress levels, and depressive symptoms of those with and without IBS were analyzed using chi-square tests and t-tests. To reduce bias from sample distortion [27] between the with and without IBS groups, the 1:4 propensity score matching method was used after controlling for demographic characteristics such as age, education level, and marital status. The influences of IBS on stress and depressive symptoms were analyzed using multivariate ordinal logistic regression analyses. In Model 1, IBS was included to determine its effect on stress and depressive symptoms. In Model 2, demographic characteristics of age, education level, marital status, BMI, alcohol consumption, and shift work were included as confounders. In Model 3, dietary habits of frequency of meals, amount of meal, and frequency of skipping meals were included. In Model 4, sleep quality and depressive symptoms or stress were included.

### 2.5. Ethical Considerations

This study was conducted after obtaining approval from the Institutional Review Board at Ewha Womans University, Seoul (Approval No. 117-4). The study participants confirmed the research purpose, voluntariness, and confidentiality and signed an electronic informed consent form before participating in the online survey.

## 3. Results

### 3.1. Prevalence and Characteristics of IBS

Among the 7667 female nurses in the 7th KNHS survey, 178 (2.3%) were classified as having IBS based on the Rome IV criteria. The IBS-related characteristics are described in Table 1. Half of the participants with IBS (49.4%) reported a decrease in the number of defecations or difficulty defecating. The most frequently used medication for intestinal health was probiotics (40.9%), followed by laxatives (12.1%) and oral antidiarrheal treatments (5.6%). The most frequently adopted health behaviors were sufficient water intake (34.4%) and probiotic intake (30.9%).

### 3.2. Differences in Groups with and without IBS

Participant characteristics are described in Table 2. After propensity score matching, we analyzed data from 890 participants (178 with IBS and 712 without IBS). The participants with IBS were 33.84 ± 5.67 years old. Half of the participants (51.7%) with IBS had a normal BMI, and 63.6% of those without IBS had a normal BMI. Approximately half of the participants were working shifts, and most participants had two meals a day (IBS: 68.5%, without IBS: 64.3%). The scores for sleep quality, stress, and depressive symptoms differed significantly between those who had IBS and those who did not. Participants with IBS had poor sleep quality (χ^2^ = 22.304, *p* < 0.001), increased stress levels (t = −6.203, *p* < 0.001), and more depressive symptoms (t = −7.282, *p* < 0.001) than those who did not have IBS.

### 3.3. Influence of IBS on Stress Levels

We conducted a multivariate ordinal logistic regression to identify whether having IBS was an influential factor for increased stress levels (Table 3). Model 1 considered only the IBS variable. Female nurses who had IBS were 2.214 times more likely to experience high stress levels than those who did not have IBS (CI: 1.579–3.104). In Model 2, demographic factors were included, and Model 3 additionally included dietary habits. The influence of having IBS on stress remained significant in Model 2 (OR = 2.158, CI: 1.529–3.044) and Model 3 (OR = 2.142, CI: 1.512–3.034). In Model 4, sleep quality and depressive symptoms were added, and having IBS was no longer statistically significant factor for increased stress levels. Among the confounding variables, having meals twice a day instead of more than three times a day (OR = 0.678, CI: 0.463–0.995), poor sleep quality (OR = 2.358, CI: 1.658–3.352), and having depressive symptoms (OR = 11.172, CI: 7.009–17.810) were significantly associated with high stress levels among female nurses.

### 3.4. Influence of IBS on Depressive Symptoms

Multivariate ordinal logistic regression was conducted to examine the influence of having IBS on depressive symptoms (Table 4). The IBS variable was inserted in Model 1. Female nurses who had IBS were 3.181 times more likely than those without IBS to experience depressive symptoms (CI: 2.178–4.647). In Model 2, demographic factors were included, and Model 3 additionally included dietary habits. The influence of having IBS on depressive symptoms remained significant in Model 2 (OR = 3.201, CI: 2.163–4.735) and Model 3 (OR = 3.124, CI: 2.096–4.656). In Model 4, sleep quality and stress level were added, and the influence of having IBS on depressive symptoms remained significant (OR = 2.205, CI: 1.361–3.574). Among the confounding variables, having large meals rather than adequate meals (OR = 1.852, CI: 1.135–3.023), poor sleep quality (OR = 4.147, CI: 2.522–6.819), and high stress levels (OR = 11.934, CI: 7.396–19.256) were significantly associated with experiencing depressive symptoms among female nurses. 

## 4. Discussion

In this study, the prevalence of IBS among Korean female nurses based on Rome IV criteria was 2.3%. In a meta-analysis, Oka et al. [4] reported that the global prevalence of IBS diagnosed using the Rome IV criteria was 3.8%, which was slightly higher than the results of this study. However, based on the Rome III criteria, the prevalence of IBS among Korean nurses and nurse assistants was 28.0% [19], and the prevalence of IBS among Chinese nurses was 17.4% [28]. It is difficult to compare the prevalence of IBS among nurses because few previous studies used the Rome IV criteria. Van den Houte et al. [5] reported that the Rome IV IBS criteria could be very restrictive because the prevalence of self-reported IBS in the general population was three times higher than the IBS prevalence diagnosed by the Rome IV criteria. Oka et al. [4] reported that the Rome IV criteria for diagnosing IBS might be less suitable for population-based epidemiological surveys because they are more restrictive than the Rome III criteria. Therefore, to investigate the prevalence of IBS, it is necessary to consider the diagnostic criteria.

In this study, the level of stress reported by the IBS group was higher than that reported by the without IBS group, but after adjusting for depressive symptoms and sleep quality, IBS did not significantly affect the level of stress. In the study of Weaver et al. [12], the IBS group experienced a higher level of stress than the without IBS group, which is similar to the results of this study. Controversial results have been reported regarding the relationship between stress and IBS symptoms. Chan et al. [16] reported that because daily life stress in patients with IBS who have diarrhea predicted a decrease in abdominal pain and urgency of defecation, appropriate daily life stress could help to improve IBS symptoms. Edman et al. [14] found a significant relationship between perceived stress and abdominal pain in patients with gastroesophageal reflux disease and inflammatory bowel disease. However, they reported finding no significant relationship between stress and abdominal pain in patients with IBS, possibly because they did not consider symptom variability by IBS subtype. In this study, we did not consider the subtypes or symptom severity of IBS. Therefore, to investigate the relationship between IBS and stress in future studies, IBS subtypes and symptom severity should be considered.

In this study, the prevalence of depressive symptoms in the IBS group was 33.1%, higher than that reported in a meta-analysis that found the prevalence of depressive symptoms in patients with IBS to be 28.8% [8]. In this study, IBS was a major risk factor that increased the risk of depressive symptoms by 2.205 times even after adjusting for covariates. Zhang et al. [29] reported that patients with IBS were 10.45 times more likely to experience moderate to severe depressive symptoms than healthy controls. Geng et al. [9] reported that patients with IBS experienced more severe depressive symptoms than patients with inflammatory bowel disease, suggesting that in IBS, brain-gut pathway dysfunction could lead to psychological manifestations of the disease. Because nurses have a high level of depressive symptoms due to their job characteristics and high work intensity, strategies to reduce the depressive symptoms of nurses with IBS are needed. Ballou and Keefer [30] reported that cognitive behavioral therapy, hypnotherapy, and mindfulness-based therapy had physiological effects to reduce the stress response and inflammation in patients with IBS. Therefore, various adjuvant therapies may be needed to ensure the psychological health of patients with IBS.

Meanwhile, among the covariates, sleep quality was a factor associated with both stress and depressive symptoms, and 60.1% of the IBS group self-reported that their sleep quality was poor, indicating that nurses with IBS experience sleep problems. According to Lee and Choi [20], nurses had higher levels of stress and depressive symptoms than other occupational groups, and the stress and depressive symptoms of nurses were associated with sleep disturbances. The high prevalence of IBS observed in rotating shift workers could indicate a relationship between IBS and circadian rhythm disturbance [19]. Therefore, for nurses with IBS, it is necessary to include stress and depressive symptoms in their management plans as an intervention to improve sleep quality. Providing supports such as adjustments in work schedules will further enhance the effectiveness of psychological care for nurses with IBS.

There are a few limitations of this study. Because this cross-sectional study could not confirm causality, further investigation through a longitudinal study is needed. The controversial results in IBS prevalence studies using Rome criteria require additional study. Because the subtypes and symptom severity of IBS were not investigated in this study, it is also necessary to identify the relationship between IBS and stress and depressive symptoms while considering those factors in future studies. Since the participants’ responses to questions such as sleep quality and amount of meal are subjective, there might be a response bias. Also, since these questions are based on subject memory of the past month or 3 months, there might be a recall bias.

## 5. Conclusions

The levels of stress and depressive symptoms among nurses with IBS were higher than those among nurses without IBS. IBS was a factor significantly affecting depressive symptoms even after covariate adjustments. Nurses have high levels of stress and depressive symptoms due to their high work intensity and irregular work characteristics. Nurses with IBS have an even higher risk of experiencing those psychological problems, which can reduce their work efficiency. Therefore, hospital managers need to develop and provide support strategies for nurses with IBS.

## Figures and Tables

**Table 1:** Characteristics related to irritable bowel syndrome (N = 178).

Characteristics	Categories	*n*	%
Decreased number of defecations or difficulty defecating	Yes	88	49.4
	No	90	50.6
- Number of defecations (times/week) (*n* = 88)	<3	49	55.7
	≥3	39	44.3
- Soft and watery stool (*n* = 90)	Yes	50	55.6
	No	40	44.4
Medications for intestinal health *	Probiotics	88	40.9
	Laxative	26	12.1
	Oral antidiarrheal	12	5.6
	Suppository	5	2.3
	Enema	3	1.4
	Other	8	3.7
	None	73	34.0
Health behaviors for intestinal health *	Sufficient water intake	109	34.4
	Probiotic intake	98	30.9
	Sufficient dietary fiber intake	49	15.5
	Restriction of dairy consumption	19	6.0
	Sufficient electrolyte beverage intake	9	2.8
	Other	5	1.6
	None	28	8.8

* Duplicate response.

**Table 2:** Comparison between groups with and without irritable bowel syndrome (N = 890).

Characteristics	Categories	IBS (N = 178)		No IBS (N = 712)		t or *x*^2^	*p*
		*n*	%	*n*	%		
Age, years	≤29	44	24.7	182	25.6	0.102	0.950
	30–39	104	58.4	416	58.4		
	≥40	30	16.9	114	16.0		
	M ± SD	33.84 ± 5.67		33.87 ± 5.77		0.073	0.942
Education level	3-year college	41	23.0	164	23.0	0.061	0.970
	4-year college	111	62.4	449	63.1		
	Master or higher	26	14.6	99	13.9		
Marital status	Single or others	94	52.8	376	52.8	0.000	1.000
	Married	84	47.2	336	47.2		
BMI	Normal (18.5–23 kg/m^2^)	92	51.7	453	63.6	12.933	0.002
	Underweight (<18.5 kg/m^2^)	22	12.4	42	5.9		
	Overweight (≥23 kg/m^2^)	64	36.0	217	30.5		
	M ± SD	22.33 ± 4.13		22.13 ± 2.98		−0.606	0.545
Alcohol consumption	No	53	29.8	211	29.6	0.001	1.000
	Yes	125	70.2	501	70.4		
Shift work	No	86	48.3	349	49.0	0.028	0.933
	Yes	92	51.7	363	51.0		
Frequency of meals (times/day)	1	9	5.1	20	2.8	4.477	0.107
	2	122	68.5	458	64.3		
	≥3	47	26.4	234	32.9		
Amount of meal	Small	17	9.6	54	7.6	1.812	0.404
	Adequate	116	65.2	500	70.2		
	Large	45	25.3	158	22.2		
Frequency of skipping meals	Rarely	90	50.6	413	58.0	3.211	0.076
	≥1 time/week	88	49.4	299	42.0		
Sleep quality	Good	71	39.9	424	59.6	22.304	<0.001
	Poor	107	60.1	288	40.4		
Stress	Low (<75th percentile)	97	54.5	517	72.6	21.849	<0.001
	High (≥75th percentile)	81	45.5	195	27.4		
	M ± SD	20.29 ± 5.44		17.62 ± 5.07		−6.203	<0.001
Depressive symptoms	No (<10)	119	66.9	616	86.5	38.280	<0.001
	Yes (≥10)	59	33.1	96	13.5		
	M ± SD	7.84 ± 5.50		4.63 ± 4.23		−7.282	<0.001

IBS = Irritable Bowel Syndrome; BMI = Body Mass Index; M = Mean; SD = Standard Deviation.

**Table 3:** Effect of irritable bowel syndrome on stress (N = 890).

Variables	Model 1		Model 2		Model 3		Model 4	
	OR	95% CI	OR	95% CI	OR	95% CI	OR	95% CI
IBS								
No	Ref.		Ref.		Ref.		Ref.	
Yes	2.214 ***	1.579–3.104	2.158 ***	1.529–3.044	2.142 ***	1.512–3.034	1.323	0.873–2.003
Age, years								
≤29			Ref.		Ref.		Ref.	
30–39			1.158	0.806–1.665	1.173	0.813–1.693	1.195	0.783–1.824
≥40			1.025	0.595–1.765	1.000	0.576–1.735	1.392	0.753–2.571
Education level								
Master or higher			Ref.		Ref.		Ref.	
3-year college			0.963	0.568–1.632	0.951	0.557–1.624	0.897	0.485–1.660
4-year college			1.024	0.645–1.626	1.034	0.647–1.653	1.251	0.737–2.126
Marital status								
Married			Ref.		Ref.		Ref.	
Single or others			1.445 *	1.043–2.002	1.455 *	1.045–2.025	1.303	0.897–1.894
BMI								
Normal			Ref.		Ref.		Ref.	
Underweight			1.438	0.829–2.493	1.366	0.776–2.407	1.528	0.798–2.926
Overweight			1.384 *	1.005–1.906	1.263	0.910–1.753	1.145	0.786–1.668
Alcohol consumption								
No			Ref.		Ref.		Ref.	
Yes			0.978	0.705–1.356	0.962	0.691–1.338	0.865	0.595–1.259
Shift work								
No			Ref.		Ref.		Ref.	
Yes			1.427 *	1.051–1.939	1.402 *	1.025–1.917	1.233	0.863–1.762
Frequency of meals (times/day)								
≥3					Ref.		Ref.	
1					0.824	0.360–1.887	0.461	0.170–1.254
2					0.759	0.542–1.061	0.678 *	0.463–0.995
Amount of meal								
Adequate					Ref.		Ref.	
Small					1.198	0.689–2.085	0.828	0.433–1.584
Large					1.574 *	1.112–2.227	1.184	0.792–1.771
Frequency of skipping meals								
Rarely					Ref.		Ref.	
≥1 time/week					1.525 **	1.123–2.072	1.395	0.981–1.984
Sleep quality								
Good							Ref.	
Poor							2.358 ***	1.658–3.352
Depressive symptoms								
No (<10)							Ref.	
Yes (≥10)							11.172 ***	7.009–17.810
Nagelkerke R2	0.033 ***		0.061 ***		0.085 ***		0.338 ***	
χ^2^/df	20.842/1		39.715/10		55.575/15		243.874/17	

* *p* < 0.05, ** *p* < 0.01, *** *p* < 0.001; IBS = Irritable Bowel Syndrome; BMI = Body Mass Index.

**Table 4:** Effect of irritable bowel syndrome on depressive symptoms (N = 890).

Variables	Model 1		Model 2		Model 3		Model 4	
	OR	95% CI	OR	95% CI	OR	95% CI	OR	95% CI
IBS								
No	Ref.		Ref.		Ref.		Ref.	
Yes	3.181 ***	2.178–4.647	3.201 ***	2.163–4.735	3.124 ***	2.096–4.656	2.205 **	1.361–3.574
Age, years								
≤29			Ref.		Ref.		Ref.	
30–39			1.176	0.760–1.821	1.179	0.756–1.838	1.115	0.659–1.887
≥40			0.490	0.226–1.060	0.481	0.219–1.057	0.499	0.209–1.195
Education level								
Master or higher			Ref.		Ref.		Ref.	
3-year college			1.293	0.675–2.477	1.197	0.617–2.323	1.719	0.785–3.765
4-year college			0.788	0.436–1.422	0.746	0.409–1.362	0.842	0.418–1.699
Marital status								
Married			Ref.		Ref.		Ref.	
Single or others			1.647 *	1.092–2.484	1.617 *	1.065–2.456	1.245	0.764–2.030
BMI								
Normal			Ref.		Ref.		Ref.	
Underweight			1.407	0.727–2.722	1.255	0.631–2.497	1.262	0.545–2.924
Overweight			1.575 *	1.064–2.331	1.435	0.959–2.149	1.239	0.767–2.002
Alcohol consumption								
No			Ref.		Ref.		Ref.	
Yes			1.228	0.804–1.876	1.198	0.780–1.839	1.409	0.846–2.348
Shift work								
No			Ref.		Ref.		Ref.	
Yes			1.379	0.941–2.021	1.291	0.871–1.914	0.883	0.554–1.409
Frequency of meals (times per day)								
≥3					Ref.		Ref.	
1					1.694	0.696–4.125	2.232	0.773–6.448
2					1.006	0.653–1.547	1.342	0.807–2.230
Amount of meal								
Adequate					Ref.		Ref.	
Small					1.918	1.001–3.676	1.783	0.816–3.896
Large					2.118 ***	1.403–3.197	1.852 *	1.135–3.023
Frequency of skipping meals								
Rarely					Ref.		Ref.	
≥1 time/week					1.359	0.930–1.987	0.984	0.626–1.547
Sleep quality								
Good							Ref.	
Poor							4.147 ***	2.522–6.819
Stress								
Low							Ref.	
High							11.934 ***	7.396–19.256
Nagelkerke R2	0.062 ***		0.118 ***		0.152 ***		0.456 ***	
χ^2^/df	33.821/1		65.674/10		85.732/15		286.403/17	

* *p* < 0.05, ** *p* < 0.01, *** *p* < 0.001; IBS = Irritable Bowel Syndrome; BMI = Body Mass Index.

## Data Availability

The datasets generated and analyzed during the current study are not publicly available because this government data needs time for data clearing and the establishment of guidelines. The Korea National Institute of Health is planning on opening this data to the public in the future.
